# Stakeholder analysis for a maternal and newborn health project in Eastern Uganda

**DOI:** 10.1186/1471-2393-13-58

**Published:** 2013-03-04

**Authors:** Gertrude Namazzi, Kiwanuka Suzanne N, Waiswa Peter, Bua John, Okui Olico, Allen Katharine A, Hyder Adnan A, Ekirapa Kiracho Elizabeth

**Affiliations:** 1 Makerere University School of Public Health, Kampala, Uganda; 2 Iganga/Mayuge Demographic Surveillance Site, Iganga/Mayuge, Uganda; 3 Department of International Health, Johns Hopkins University Bloomberg School of Public Health, Baltimore, MD, USA; 4 Karolinska Institutet, Division of Global Health, , Sweden

**Keywords:** Stakeholder analysis, Maternal and newborn health, Eastern Uganda, Future health systems

## Abstract

**Background:**

Based on the realization that Uganda is not on track to achieving Millennium Development Goals 4 and 5, Makerere University School of Public Health in collaboration with other partners proposed to conduct two community based maternal/newborn care interventions aimed at increasing access to health facility care through transport vouchers and use of community health workers to promote ideal family care practices. Prior to the implementation, a stakeholder analysis was undertaken to assess and map stakeholders’ interests, influence/power and position in relation to the interventions; their views regarding the success and sustainability; and how this research can influence policy formulation in the country.

**Methods:**

A stakeholder analysis was carried out in March 2011 at national level and in four districts of Eastern Uganda where the proposed interventions would be conducted. At the national level, four key informant interviews were conducted with the ministry of health representative, Member of Parliament, and development partners. District health team members were interviewed and also engaged in a workshop; and at community level, twelve focus group discussions were conducted among women, men and motorcycle transporters.

**Results:**

This analysis revealed that district and community level stakeholders were high level supporters of the proposed interventions but not drivers. At community level the mothers, their spouses and transporters were of low influence due to the limited funds they possessed. National level and district stakeholders believed that the intervention is costly and cannot be affordably scaled up. They advised the study team to mobilize and sensitize the communities to contribute financially from the start in order to enhance sustainability beyond the study period. Stakeholders believed that the proposed interventions will influence policy through modeling on how to improve the quality of maternal/newborn health services, male involvement, and improved accessibility of services.

**Conclusion:**

Most of the stakeholders interviewed were supporters of the proposed maternal and newborn care intervention because of the positive benefits of the intervention. The analysis highlighted stakeholder concerns that will be included in the final project design and that could also be useful in countries of similar setting that are planning to set up programmes geared at increasing access to maternal and new born interventions. Key among these concerns was the need to use both human and financial resources that are locally available in the community, to address supply side barriers that influence access to maternal and child healthcare. Research to policy translation, therefore, will require mutual trust, continued dialogue and engagement of the researchers, implementers and policy makers to enable scale up.

## Background

Uganda is not on schedule to achieve Millennium Development Goals (MDGs) 4, 5 and 6 [1,2] though maternal child health (MCH) is one of the priority areas in the Uganda National Minimum Health Care Package. Perinatal and maternal conditions contribute 20.4% of the total burden of disease [3]. Despite high use of antenatal care (ANC) (94% for 1st ANC visit) and 70% of the population living within five kilometer distance to a health facility [4], deliveries at health facilities have remained at 57% [5] and Emergency Obstetric Care (EmOC) met need is only 14% [6]. Postnatal care (PNC) coverage is also low at about 10%. Consequently, maternal mortality ratio, perinatal and neonatal mortality rates have remained high at 438 per 100,000 live births, 36/1,000 and 27/1000 live births respectively [5]. The poor health indicators may be due to the limited health expenditure in the country [7].

Households in Uganda constitute almost 50% of the source of health financing, yet 31% of the population live below poverty levels [8]. Although the Government of Uganda (GoU) health expenditure per capita of $ 33, has improved over the past decade, it is still inadequate to address the health delivery challenges [9]. The proposed national health insurance scheme for additional funding for the health sector may however worsen the existing disparities between rural and urban areas [10]. An intermediate financing approach may be utilized to introduce the government, health providers and the general population to concepts and mechanisms of insurance. Voucher programs are one form of output based health care approach that empowers clients to choose their providers [11]. Evidence from the reproductive health voucher programs in Kenya and Western Uganda indicate an increase in utilization of services especially by the targeted populations such as the poor. However, the non-redemption of some purchased vouchers and high administration costs may hinder long term aims. Administration costs could however, be reduced by integrating them into national strategies [12]. This emphasizes the need for effective approaches that might inform rapid scale up of evidence-based interventions within the existing health system [13].

The Makerere University School of Public Health (MakSPH) in collaboration with other partners (Johns Hopkins University Bloomberg School of Public Health, USA and Save the Children) piloted two intervention studies (the Safe Deliveries Study and the Uganda Newborn Study). The studies were aimed at improving access to appropriate maternal and newborn care in rural Uganda. In the Safe Deliveries Study pilot, vouchers for services and transport for delivery care were used to increase institutional deliveries from <200 to 500 deliveries per month [14]. The Uganda Newborn trial (UNEST) used Community Health Workers (CHWs) to link communities and facilities for improved maternal and newborn care. Early implementation experiences showed high acceptability of CHWs, antenatal care (ANC) attendance and facility deliveries [15]. Both projects demonstrated intervention strategies that are quickly adopted, generated large demand, and showed a rapid impact on deliveries and other maternal and newborn care health services. However, key knowledge gaps include how to integrate these packages into the routine health system, how to implement them in much larger populations, and how to make their funding sustainable.

Using the experience from the pilot studies, Makerere University School of Public Health with support from the World Health Organization (WHO) and the Future Health Systems (FHS) consortium (http://www.futurehealthsystems.org), proposed two community based maternal/newborn interventions entitled “*Innovations for Increasing Access to Integrated Safe Delivery, PMTCT and Newborn Care in Rural Uganda*” (MANEST) and “*Maternal and Neonatal Implementation for Equitable Systems*” (MANIFEST). MANEST is a 30 months study, to be implemented in three districts in eastern Uganda. We proposed three strategies: Health facility strengthening through mentoring of frontline health workers for maternal/newborn care; operationalising Village Health Teams (VHT) (essentially community health workers) to increase community awareness for improved birth preparedness and health seeking behavior; and use of transport vouchers to improve access to institutionalized deliveries. Two transport voucher options were proposed. These included: fully paid vouchers that would benefit only the poor or partially paid vouchers that would target all pregnant women. The partial payment was estimated at 2–3 USD for the return journey from the health facility. Similar strategies were proposed under MANIFEST, except that there are no transport vouchers. Community mobilization strategies will be implemented for three years to increase awareness about maternal and newborn care, promote linkages with financial social networks and local transporters to improve financial preparedness and timely transport use. The two proposed projects intend to generate evidence that will contribute to the development of sustainable community based programmes that can be scaled up, in an attempt to increase access to integrated maternal and child health services.

Research quality, as well as its impact on policy and implementation, is likely to be enhanced when multiple stakeholder perspectives, particularly those that are consumer derived from real life situations, are taken into consideration, [16,17]. Stakeholder analyses are useful for identifying key stakeholders, assessing their knowledge about the problem identified for intervention, their interests, power/policy influence, alliances and the importance that they will attach to the project [18]. Furthermore they allow the examination of the individual, group and institutional landscape amongst relevant stakeholders, their relationships and the issues they care about most and how these may affect the project [19,20]. The engagement of stakeholders is therefore critical for the success of every project or programme. They should be engaged from the outset during problem definition and project design, since the decisions and actions taken in a project affect them. Their participation ensures that legitimate stakeholder interests and concerns are effectively addressed [21].

In order to ensure that the proposed projects address stakeholder concerns a stakeholder analysis was undertaken. The specific objectives of the stakeholder analysis included: 1) to assess and map stakeholders’ interests, influence/power and position in relation to the proposed community based maternal/newborn interventions; 2) to assess stakeholders’ views regarding the success and sustainability of the intervention beyond the study period; and 3) to inform the refinement and implementation strategy of the community based maternal/newborn interventions. This paper reports on the process and results of this stakeholder analysis with the recognition that we hope to repeat such an analysis after the first year of project implementation.

## Methods

### Setting

The stakeholder analysis was carried out in March 2011 at the national and district level in Uganda. Local level participants came from four districts of Buyende, Kamuli, Iganga, and Pallisa where the study projects will be conducted.

The stakeholder analysis was undertaken through three major phases 1) Identification of stakeholders, 2) Assessing and mapping out their interests and attitudes, power/influence, position in relation to the political resources they possess [22], and 3) Development of an appropriate strategy on how best to interact and engage these stakeholders.

### Identification of stakeholders

This step involved brainstorming amongst the research team members to identify categories of stakeholders, how they may be affected, who were likely to be direct beneficiaries, the potential impact of the project upon stakeholders, and their numbers. A list of categories of stakeholders was then prioritized based on: potential to benefit, weaken or strengthen the intervention; at national, district, and community level (Table 1) within 11 major categories.

**Table 1:** Stakeholders identified and interviewed for the Uganda project

**Level**	**Stakeholders**	**Number of interviews**
National	Ministry of Health	1
	Member of parliament	1
	Development partners	1
	Religious bureaus	1
District	District leadership	2
	District Health Team	2
	District Health Team	Workshop
Health sub district	Health providers	
	Public providers	2
	Private providers	2
Village	Households	
	• Women	4 FGDs
	• Men	4 FGDs
	Opinion leaders	
	• Community leaders	4 IDIs
	• Transporters	4 FGDs

*FGDs* = Focus group discussion, *IDIs* = Key informant interviews.

### Data collection methods

Data were collected from the respondents indentified in Table 1. Data collection techniques involved mainly qualitative methods and these included: Focus group discussions with mothers and their spouses, and transporters; Key informant interviews with national level participants, community leaders, district health team members and administrators; and a stakeholders’ workshop with 12 representatives from the district health teams from the districts of Buyende, Kamuli, Iganga, and Pallisa. Data were collected by researchers with experience in qualitative data collection techniques. National and district level interviews were conducted in English while at the community level the interviews and discussions were in Lusoga, the local language. Two *Lusoga* speakers independently translated interviews into English. All interviews were digitally recorded and transcribed.

#### Key informant interviews

Key informants were purposively selected and key informant interview guides were used to collect data from 4 national, 8 district and 4 community level representatives as shown in Table 1. Questions and discussions focused on topics such as factors affecting utilization of maternal child health services, strengths and weaknesses of the proposed study, how integration into existing health and community services can be accomplished, challenges, potential solutions, and sustainability strategies.

#### Focus group discussions

Focus group discussions (FGDs) were conducted to explore opinions, attitudes and perceptions on the feasibility of implementation, and sustainability of a maternal/newborn care research project at the community and household levels [23,24]. Participants included mothers and their spouses (men). Four FGDs of women and four FGDs for men in the intervention areas of Buyende and Pallisa, and the comparison areas of Kibuku and Kamuli were conducted. The discussion with mothers who are the program’s primary beneficiaries focused on barriers to service utilization [25,26], opinions regarding the proposed intervention, how the intervention can successfully be integrated into existing structures, potential implementation challenges and sustainability strategies.

Transporters (motorcycle riders) were also interviewed to seek their views, the challenges they face and possible solutions for the use of transport vouchers for maternal and newborn services. Two focus group discussions were held with transporters in the intervention area and two focus group discussions with those in the comparison area.

The participants in all the FGDs were purposively selected. The groups were homogenous in composition (mothers aged 18–35 years, men aged 18–35 years, and transporters aged 35 – 50 years). The discussions were guided by an FGD guide and focused on challenges in transporting mothers, information the transporters would like to share, means of communication and possible community contributions for sustaining the scheme.

Ethical approval to conduct the study was provided by the Institutional Review Board at the Makerere University School of Public Health and Uganda National Council for Science and Technology (UNCST). Voluntary informed consent was then individually obtained from all the study participants.

### Identifying stakeholders power, position and influence

**Figure 1:**
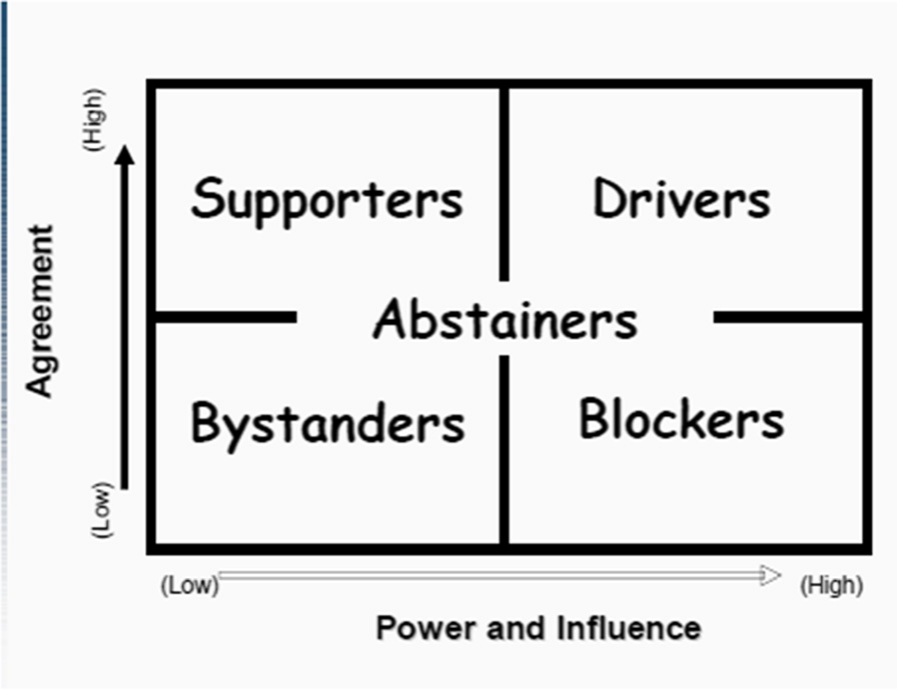
**Stakeholder analysis grid. ***Ref: FHS2/Stakeholder Analysis/Hyder et al.*[27].

Assessing and mapping the power/influence of the stakeholders involved identifying who owns what resources (tangible or intangible), who possesses privileges, and who can directly or indirectly take action for or against the project or be able to mobilize for or against it. This assisted in the organization of stakeholders according to their likely influence over decisions to be made, and the likely impact of project decisions upon them. During this phase a stakeholder analysis grid was formulated and the stakeholders were characterized according to the following pre-existing categories as predicted in Figure 1. The categorization was based on the interview findings, the positions they held, and previous roles they played in the pilot studies.

Drivers: A person or group that has high influence and champions the cause

Blockers: A person or group that has high levels of power, but opposes the proposal

Supporters: Those that support the proposal, but whose influence may be limited (on their own)

Bystanders: Those with low influence and support.

Abstainers: Those who are neutral to the proposal, but may or may not have influence.

The classification of the stakeholders was further categorized as high, moderate or low, considering their varied levels of alignment and influence. A table was used to present the findings since most of the stakeholders were supporters (same category of the grid), secondly this allowed the inclusion of other results and strategies to deal with the stakeholder. Such strategies were classified into:

(1). Empowerment: Strategies that would economically empower communities, encourage saving or raise awareness

(2). Continuous engagement: Keep the stakeholders updated of the implementation process in order to take action

(3). Involve further: Engage in planning or implementation

(4). Consult further: Agree on working relations

### Thematic analysis

Thematic analysis of interviews and mapping of stakeholders by their level of power, influence, and level of agreement was conducted. The researchers, who are considered internal analysts [21] led this process. Although we used a framework that highlighted the key areas of investigation, we also looked out for new themes emerging outside this framework [28,29]. The themes identified were in line with the key issues that the research sought to address, such as concerns and interests of stakeholders, sustainability challenges and suggestions for promoting sustainability and uptake of the research findings.

## Results

### Power/influence and position of stakeholders

All the stakeholders interviewed were found to be in agreement with the proposed study, although there were varying levels of power/influence. On the whole, community level stakeholders were found to be supportive of the intervention but most of them had little influence due to the limited resources under their control.

Pregnant and newly delivered mothers were found to be highly in agreement of the proposed study (Table 2). In general, mothers applauded the intervention given that it offered them an opportunity to deliver at health facilities at the hands of skilled birth attendants. These thoughts are captured in one of the quotations below from one of the participants of a focus group discussion.

“....*long ago we used to deliver from banana plantations and on the way which made us get problems while giving birth. So it saved us from all those problems, we are now very happy, let the project continue.”* [FGD women]

**Table 2:** Stakeholders characteristics, power/influence regarding the implementation of an integrated maternal/newborn care project

**Stakeholder**	**Characteristics**					
	**Interest in the issue**	**Current level of power/influence**	**Type of power/leadership**	**Current level of agreement with proposal**	**Classification of the stakeholder (on a three point scale)**	**Strategy to deal with the stakeholder**
**Mothers**	Pregnant and newly delivered women	Little influence	Beneficiaries	Strong Agreement	High level supporter	Empower
**Men**	Men make most of the decisions in the family including when and where to seek for health care	Little influence	Beneficiaries	Strong Agreement	High level Supporter	Involve further and empower
**Transporters**	These are motorcycle riders who ferry passengers (mothers) at a fee in form of a voucher	Moderate influence	Transporters/Beneficiaries	Strong Agreement	High level Supporter	Involve further
**Local Council member (LC)**	The local council is the administrative structure of the community	Little influence	Opinion leader	Strong Agreement	High level supporter	Involve further
**Health providers**	Health workers within the Health facilities both private and public	Moderate influence	Beneficiaries	Strong Agreement	High level supporter	Involve further
**District Health Team (DHT)**	The DHT members are in charge of/supervise health matters in the district	High influence but limited resources for implementation	Decision makers	Strong Agreement	High level supporter	Involve further
**Member of parliament (MPs)**	Woman MP	High influence for policy formulation	Decision maker	Strong Agreement	High level supporter	Involve further
**Senior Medical Officer MOH**		High influence for policy formulation but limited funds for scale up	Decision maker and influences policy formulation	Moderate Agreement	Moderate level supporter	Continuous engagement
**Development partner**	Donor	Moderate Influence of implementation	Funder	Strong Agreement	High level supporter	Continuous engagement
**Uganda Catholic Medical bureau**	Regulates policies regarding management of Catholic faith based facilities	High influence of catholic based facilities, moderate influence of intervention	Catholic faith is widely spread in the country with many faith based facilities	Moderate agreement	Moderate level supporter	Consult further

However, due to the limited resources mothers were found to control, their power and influence was considered to be low, hence they were characterized as supporters (Figure 1).

During the FGDs, men revealed that they support the programme strongly and would love to see it continue for a longer time. The burden of paying for transport costs, buying the supplies and requirements for the mothers and their newborn babies usually rests on the men as household heads. Financial contributions from the project will therefore enable them to reallocate their meager resources towards other basic pressing needs such as school fees, food and general welfare of the family. The men were also characterized as supporters due to their high level of agreement with the intervention but rather limited influence.

The level of support of the motorcycle riders, who were involved in the transport business and had been engaged by the existing safe delivery study, was also analyzed. Since they transported pregnant and newly delivered mothers for a fee, they were also considered beneficiaries. This group was very supportive of the maternal/newborn study project. These transporters were found to have moderate influence since their participation is critical for the successful implementation of the transport voucher scheme.

The local council (LC) which is the administrative structure of the community starting with the village (LC 1), the parish (LC 2), the sub-county (LC 3) and the district level (LC 5) had varying resources; least at the lowest level with marginal increases as the level of administration rises. The local leaders were eager to get involved in the implementation of the study, especially through mobilization and sensitization of the local community. They were cognizant of the challenges mothers face in the community such as high poverty levels and they welcomed the study project. The quote below highlights their views.

“….*the money they (mothers) have been paying in the hospital was a burden to them and even transport, so if the project comes in, then there will be no problem.”* [LC 1 women representative]

Table 2 summarizes the stakeholders’ classification regarding the power and influence towards the planned study project.

At the district level, one of the technical departments is health, which is run by the District Health Team (DHT) headed by the District Health Officer (DHO). The DHOs realized how their communities and health facilities were likely to benefit, hence they strongly approved of the intervention, as illustrated in the quote below.

*“..Our community will truly benefit and also at the end of the day our health workers will be trained, which we probably cannot afford as a district to keep training them. I think am happy, I have a strong go ahead for it. I only see a challenge of sustainability.”* [District Official]

The DHOs had high influence; they are in charge of the district’s health care system and they approve health interventions that are implemented in the districts. However, due to the limited resources they could commit to the implementation of the intervention they were characterized as supporters rather than drivers.

Level of agreement with the proposed maternal/newborn care study for the various national level stakeholders was analyzed and was found to be predominantly high for most of the stakeholders. However, the MOH representative was skeptical of the success and sustainability of the proposed interventions given the ‘high’ costs of implementation of the transport voucher scheme as emphasized by the following quote.

*‘..every research is to inform policy but you are putting in a lot of resources, you are using a lot of money which the government cannot afford. You can’t sustain it….it becomes a very big issue, the government will not put money as direct as you are doing….the way you are doing it’*. [MOH official]

The MOH representative provides oversight over health activities and programmes in the country, but since MOH had limited funds for scale up they were characterized as supporters.

The Member of Parliament is a policy maker and therefore key for influencing budget allocations. The development partners, such as DFID who funded the Safe Deliveries Study, indicated a desire to be involved in the research to policy translation process, and to share the research findings with other development partners at various forums. However the agent was found to be with moderate influence in the implementation process, hence a supporter and not a driver.

#### Implementation challenges and solutions highlighted by the stakeholders

The stakeholder analysis also aimed at assessing the stakeholders’ views regarding potential challenges and strategies for sustaining a transport voucher scheme, a health worker incentive scheme and strong community engagement. There were varied suggestions in this regard as summarized in Table 3.

**Table 3:** Implementation challenges and solutions suggested by stakeholders in Uganda

**Stakeholder**	**Challenges**	**Potential solutions**
Community level stakeholders	- Lack of follow up of clients	- Strengthen supervision to ensure follow up of clients and effective use of funds
	- Inadequate sensitization of beneficiaries	- Sensitize communities/TBAs, women groups/men
	- Poor quality health services (Lack of medicines, few health providers, rude nurses, unofficial payments for supplies),	- More equipment and supplies in facilities and provide an ambulance or voucher for a taxi to take patients to higher levels
	- Problems with referral transport and low male involvement	- Extend voucher scheme to other sick people, rather than pregnant mothers alone
	- Poor infrastructure	- Male involvement e.g. through escorting their wives to facilities especially at night, and financial contribution
District level stakeholders	-Sustainability of the intervention	- Sensitize community through community leaders (to mobilize the men to take part in reproductive health care)
	- Lack of required infrastructure, resources	- Sensitize and build capacity for health workers to provide quality services in an integrated manner
	– supplies, equipment and health workers	- Add ambulance services to supplement the motorcyclists.
		- Communities should contribute finances
**National level stakeholders**	-Sustainability of the project	- Have discussions with stakeholders (Members of Parliament/speaker/ministry of health, etc.) to promote implementation of project
	- Lack of required infrastructure, resources	- Implement through existing systems e.g. the VHT
	– supplies, equipment and health workers	- Conduct implementation research based on MOH strategic plan
	- Paying transporters enough	- Collaboration with developmental partners, line
	money so that they continue their work actively	ministries and local council members to improve the infrastructure
		- Regular meetings between project implementers and other stakeholders

The concerns of the community stakeholders ranged from, issues that contribute to the poor quality of services, poor access of transport services especially for referral to higher levels of care, low male involvement as well as implementation challenges experienced during the Safe Deliveries project. The district and national level stakeholders had similar concerns in addition to ensuring the sustainability of such a scheme beyond the study period. Some of the concerns raised are expounded in the sections that follow with selected quotations.

### Quality of health care services

The stakeholders pointed out that the poor quality of maternal health care services has for long been one of the deterrents to maternal and child health service utilization. The poor quality of services had previously been attributed to problems such as unofficial payments, the poor attitude of health care workers, insufficient supplies, equipment and medicines among others.

*“…. the poor attitude of health workers, then lack of supplies because if a mother comes to the facility and you give her an endless list of supplies to buy, she will end up going home because she doesn’t have the money to buy all those things”*. [District Official]

One of the districts shared how they dealt with drug stock outs. This district attempted to redistribute drugs centrally to avoid stock outs in some high catchment facilities and managed over stocking in facilities that were underutilized. Other strategies recommended for overcoming the challenges included capacity building of health workers coupled with supportive supervision.

### Transport and referral system

It was noted that the districts had a poor road network. The main mode of transport in the community was the commercial motorcycle (*boda bodas*). These motorcycles were also used to refer mothers to higher-level facilities since most of the lower level health facilities either did not have an ambulance, or had one that was not functional. Patients were therefore often required to meet either the cost of fuel if an ambulance was available or the entire cost of hiring a vehicle to a referral facility. This made it impossible for some clients to receive health care. These difficulties experienced in accessing transportation from home to health facilities and during referrals featured prominently as one of the concerns for most stakeholders.

*“…..we have no transport to bring our expectant mothers from deep villages to trading centers where there is transport, so that’s why we get so many complications during delivery; that’s when mothers produce babies who are tired and at times they produce dead babies due to lack of transport.”* [FGD men]

*“Transport in this whole district is still a challenge. The ambulances are there but there is no fuel. The ambulances are there and the driver is not facilitated. So I think we have to look into how to incorporate all this. Even the ambulances are very old. They frequently breakdown and the maintenance cost is very high because they are very old.”* [District Official]

### Male involvement

Most of the district and community level stakeholders felt that male involvement was rather low. This is depicted in the quotation below.

*“… Most of our women here in our service area are poor and you have a challenge of male involvement. I really don’t know how we can address that challenge so that we bring the male counterparts on board, so that they come to assist their spouses…. ….”* [District Official]

Some stakeholders (women) felt that men had become “lazy” because the project was assisting and providing most of the financial contributions that they were supposed to provide. This could be a potential negative consequence of the transport voucher scheme of the maternal/newborn project and thus may affect the future contribution of households towards birth preparedness.

The DHO’s recommended an intervention that will educate the men about their primary responsibility in caring for their spouses, and one that would compel them to save money and contribute to the scheme for birth preparedness.

“….*Sensitize the community well so that men know they should take care of their spouses.”* [District Official]

The district stakeholders also suggested giving mothers who come to health facilities with their husbands *maama* kits (kit of items for delivery) to encourage male involvement. One of the districts shared their experience in which they initiated a project to identify “model families.” A model family is one where critical family support for child survival is given. Such a family provides the basic family needs, has food security, practices family planning, and the husband supports his wife by accompanying her to the facility for maternal/child health care. Model men were used for peer to peer education on health issues such as family planning and service utilization. District officials even recommended passing a ‘by-law’ to enforce male involvement in birth preparedness as an important issue to consider.

The district stakeholders also proposed engaging local leaders and drama groups to sensitize communities on service utilization and birth preparedness.

### Transport voucher scheme implementation challenges

Community members shared their experiences during the implementation of the transport voucher scheme of the Safe Deliveries project. They complained that there was high inflation, consequently fuel prices increased dramatically while the transportation voucher charges remained constant. This affected the participation of some transporters and subsequently reduced transport availability in remote areas. These stakeholders recommended an increase in the transport voucher payment rates and regular review of the voucher charges.

The transporters also reported that in some cases they incurred losses. In some circumstances, they arrived and found that mothers had already delivered. In another scenario they were not paid if the health workers did not register the names of the mothers at the facility (they were only paid for clients whose names were found registered in the facility registers). Stakeholders recommended stamping and signing of the transport vouchers by health workers once a mother has been transported to the facility. Delays in payment and insecurity at night were some of the other challenges pointed out. The use of bank accounts was suggested as a means of reducing the delayed payment. Encouraging male involvement where husbands escort their wives to health facilities and the mobilization of community leaders were recommended in order to mitigate problems related to insecurity at night.

In a few cases, the mothers were not able to reach the health facility and the transporters had to assist them in delivery. Although this was a rare occurrence, the transporters suggested that they should receive emergency supplies such as gloves and basic training on how to deal with such emergencies.

### Accountability of financial incentive

It was noted that both financial and non-financial incentives for health workers were inadequate. Monetary incentives for health workers were welcomed; however, they have the potential to create rifts between those who receive such benefits and those who do not. National level stakeholders suggested conditional transfer of funds and performance based bonus payments to health facilities that can be utilized for motivation and purchasing needed materials. The stakeholders recommended strengthening of supervision of the voucher scheme to ensure that redeemed service funds at the health facilities are well utilized.

### Sustainability issues

National level stakeholders, particularly the MOH showed moderate support for the proposed intervention. As already pointed out in the previous sections, MOH stakeholders were particularly concerned about the capacity of the Ministry to scale up the initiative that appeared rather expensive. However, other national level stakeholders (development partners) noted that if the study finds the program to be cost-effective, then the government may be convinced to allocate money to scale up the project.

Most of the suggestions for improving the implementation of the scheme and promoting sustainability revolved around the increased involvement of a wide range of stakeholders. These stakeholders could provide for the successful implementation of the scheme in three main ways: by contributing finances, contributing non monetary resources, and promoting research to policy influence. Most participants highlighted the importance of engaging stakeholders at various levels to increase their awareness and encourage their participation in the project.

### Community contribution of finances

The stakeholder analysis also revealed that the national and district level stakeholders wish to see the mothers/communities take responsibility of their own health by contributing financially to the transport scheme. During the interviews it was mentioned that monetary contribution to the project has the potential to improve ownership and to minimize abuse of the vouchers. They also stated that for communities to contribute to the scheme they need to be empowered economically. The opinions of stakeholders about impact on the poor were mixed; some stakeholders acknowledged the presence of poor families and advised their exemption from transport payments. They suggested that the community members should be profiled in order to identify and exempt the poor, as described by the following quote.

*“……Government isn’t going to be able to afford services with the current economic trend. If there is a mechanism through which people can pay or contribute this would help. Under such a scheme, the poor should be exempted”* [National level KI].

However, other stakeholders such as Member of Parliament pointed out that if community members were to contribute, a flat fee should be set for everyone rather than exempting the poor or having a sliding scale with the poor paying less than the rich. They argued that one of the difficulties with a sliding scale would be to identify and determine who is poor given the subtly different poverty demarcations.

The stakeholders recommended that although the project design should include mechanisms to ensure community contribution and ownership from the start, the government should commit to saving mothers and their newborns. These thoughts are captured in the quotation below

*“The study members should keep these stakeholders informed and engaged through consultation but also they must be made to understand that saving mothers and their newborns cannot be achieved without a cost; the programmers and government must be ready to invest in inputs for improving access and health facility strengthening.”* [National level KI]

### Mobilizing resources from other partners

The district authorities recommended that the project liaises with stakeholders like the ministry of transport and non-governmental organizations to advocate for the provision of vehicles that could be maintained more easily. They suggested the purchase of motorcycles that could be used by the district health team for supervision and motorcycle ambulances for referrals to more distant health facilities.

The districts also shared that it was possible to lobby for resources from other partners working within the district. For example, two of the districts had lobbied and obtained funding for purchasing registers and training village health teams from an NGO partner working in the district.

### Research to policy influence

The stakeholders thought that areas where this study could influence policy formulation included: devising strategies for male involvement, improving the capacity of health facilities to deliver quality services, and improving the referral system especially the functionality of ambulances to enable patients reach higher levels of care.

The main problem anticipated to be encountered when trying to promote the translation of the research findings into policy; would be the high cost of implementation of the project vis a vis the outcomes and how to ensure sustainability.

“..o*f course there is going to be noise about cost. That is going to be the number one. Despite the fact that the policy makers will have the will, the cost remains a constraint”.* [District Official]

To overcome the barriers related to the translation of research to policy, stakeholders advised the project management to: bring several partners on board (parliamentary social service committee, MOH representatives and development partners); implement through existing structures; conduct implementation research based on MOH strategic plan; and have continued engagement between researchers, implementers and policy makers through meetings, policy briefs, and regular reports.

## Discussion

This study, which assessed and mapped stakeholders’ interest, influence/power and position in relation to the proposed community based maternal/newborn interventions, revealed that almost all the stakeholders supported the proposed intervention. This is an important factor in determining the success of the intervention, [30,31] which could make mobilization and buy-in easier. The high level of support and agreement could be linked to the potential benefits of the intervention. This was appreciated by the direct beneficiaries and also indirect beneficiaries such as local and district leaders who were glad to see their community benefit. Another possible reason is related to the fact that the stakeholders were consulted during the design phase of the pilot projects and their suggestions were incorporated into the programs.

The lack of drivers could be attributed to the way the original two projects were designed. Although the local stakeholders were involved, a significant amount of the implementation was done by the project. Key implementation decisions were also made by the project. Drivers with high influence at the district and community level are important for ensuring sustainability of programs. At national level, drivers who have high influence can be instrumental in increasing policy influence and scale up [18].

Stakeholders such as men, local community leaders and the district health team need to be shifted from being supporters to drivers. This could be done through a combination of methods that involve community sensitization, empowerment and integration into existing district systems during program implementation. Men wield a lot of power in Ugandan households, since they are often the family heads, custodians of family finances and the key decision makers [32]. However, male involvement in maternal and newborn health care is still limited [33]. Further engagement by the study team is important to ensure that men remain aligned and interested in the scheme, since they play a key role in deciding whether or not their households will contribute to the scheme. There is therefore need to sensitize and mobilize them to actively provide financial contributions and participate in birth preparedness. The local leaders can also play a key role in raising awareness about maternal and child health and encouraging the community to participate in the program. The existing community resource identified from the analysis that can contribute effectively towards the mobilization and the sensitization is the local council structure. All the LC representatives who were interviewed were willing to support the project. The study team should therefore equip them with the appropriate knowledge, engage and involve LCs further to mobilize the community, sensitize on respective roles and advocate for the households to contribute towards the project.

Some of the national and district level stakeholders felt that the costs of voucher implementation are beyond what the MOH can afford. In order to integrate the study project in the existing health systems, the study team should engage and involve the MOH and district officials further in planning, implementation and supervision of the intervention. They should be kept updated of the intervention progress so that they can address issues of policy concern and mobilize funds to commit to scaling up. This would foster ownership of the programmes by national and district level stakeholders [11] i.e. promotion of the DHTs and MOH from not just being supporters but drivers of the study project and prevent them from becoming blockers. Further discussions between the implementers, districts, communities, ministry of health, and ministry of finance may be useful in ensuring that the study findings can be scaled up.

Some of the key knowledge gaps identified during the pilot of the UNEST and Safe deliveries studies included: how to integrate these research project packages into the routine health system, and how to make their funding sustainable [34]. Further involvement of the community in generation of financial resources and implementation of the program is likely to promote sustainability.

Since both national and district level stakeholders are in agreement about the need for households to contribute to the costs of their health care, it provides a platform for discussion of how to empower households economically [35]. However, during the design phase of projects, attention should be given to ensuring that the poor are not excluded from receiving care. Furthermore identification of the poor should be done diligently to avoid rifts and exploitation within the community. The project will encourage partnerships and will explore devising innovative means of empowering households economically, so that they can save money to contribute towards birth preparedness and transportation to health facilities. The issue of financial contributions to health care by households in low income countries is an area that requires further research especially in countries where health insurance is being considered.

Regarding research to policy influence, the study project has the potential of influencing policy on how to improve quality of health services through modeling the continuous re-training of health workers, their motivation, service provision and supplies, as well as mechanisms of community involvement and accessing health services. The foreseen barrier to influencing policy and scale up is mainly the high costs of conducting the intervention. Research to policy translation will require mutual trust between the researchers and the policy makers [36,37], continued dialogue and engagement throughout the implementation of the study project so that timely and appropriate research findings can inform policy and scale-up interventions [38].

Lessons from this stakeholder analysis are useful for informing the implementation of the proposed programs and other maternal and newborn health interventions in countries of a similar setting. Consultation of stakeholders during the design phase and inclusion of their ideas promotes support and agreement of interventions. Inadequate involvement of the local stakeholders in implementing the programs on the other hand may limit the number of drivers who could play key roles in promoting scale up and sustainability. Community sensitization and mobilization for contribution towards the project, male involvement, and implementation through existing structures would foster ownership which is critical for any project success and scale up. Also important to emphasize is strengthening of health facilities for improved quality of care. The design phase should include plans to address ‘supply’ side barriers. These should include strategies for improved referral transportation, training of health workers, supportive supervision, provision of supplies, and sensitization.

### Methodological considerations

Although we tried to reach several categories of stakeholders who might influence the intervention, we were unable to interview some key stakeholders like ministry of finance representatives and those involved in the voucher scheme elsewhere in the country. Another limitation of this study is that the views of organizational representatives interviewed may not be generalizable to the entire organization. Further still the views of stakeholders may change with time. However, despite the inherent limitations, stakeholder analysis is a vital tool in informing the design of any form of health systems research or intervention.

## Conclusion

This stakeholder analysis has revealed that most of the stakeholders at district/community level are high-level supporters of the proposed integrated maternal newborn care package. The proposed intervention should ensure active involvement of local stakeholders in the implementation of the projects so that they can move from being passive supporters to active drivers of the work in Uganda. Research to policy translation, therefore, will require mutual trust, continued dialogue and engagement of the researchers, implementers and policy makers to enable scale up. Conducting stakeholder analysis is an important starting point for the project design in view of incorporating stakeholders’ concerns. The study findings generated add to global knowledge particularly for countries with similar settings that are planning to set up maternal and newborn interventions or any other intervention aimed at increasing access to health services.

## Competing interests

The authors declare that they have no competing interest.

## Authors’ contributions

GN, EEK, SNK and OO developed the concept for the study, participated in the design and tools development. EEK is the Ugandan PI for the Future Health Systems study team. AH, and AK came up with the analysis plan, contributed to the formulation of the study, reviewed and provided substantial inputs into the manuscript. GN, EEK, SNK, PW, JB did field work, performed the analysis reported in this paper and co-wrote drafts of the manuscript. All authors read, provided substantial input and approved the final manuscript. GN and EEK are guarantors of the paper.

## Pre-publication history

The pre-publication history for this paper can be accessed here:

http://www.biomedcentral.com/1471-2393/13/58/prepub
